# Comparative study on the curative effect of laparoscopic nephron sparing surgery and renal functions under selective segmental renal artery clamping and main renal artery clamping

**DOI:** 10.12669/pjms.36.2.1505

**Published:** 2020

**Authors:** Yuan-hua Liu, Hai-tao Dai, Chang-mao Liu, Zhong-yu Wang, Jiang Zheng

**Affiliations:** 1 Yuan-hua Liu, Department of Urology, Jingzhou First People’s Hospital, Jingzhou, 434000, Hubei, P.R. China; 2 Hai-tao Dai, Department of Urology, Jingzhou First People’s Hospital, Jingzhou, 434000, Hubei, P.R. China; 3 Chang-mao Liu, Department of Urology, Jingzhou First People’s Hospital, Jingzhou, 434000, Hubei, P.R. China; 4 Zhong-yu Wang, Department of Urology, Jingzhou First People’s Hospital, Jingzhou, 434000, Hubei, P.R. China; 5 Jiang Zheng, Department of Urology, Jingzhou First People’s Hospital, Jingzhou, 434000, Hubei, P.R. China

**Keywords:** SSRAC, MRAC, LNSS, T1 localized renal tumor, Warm ischemia, Renal function

## Abstract

**Objective:**

To discuss the clinical effect and safety evaluation of laparoscopic nephron sparing surgery (LNSS) under selective segmental renal artery clamping (SSRAC) and main renal artery clamping (MRAC).

**Methods:**

Eighty-four patients with T1 localized renal tumors who were admitted and treated from October 2017 to October 2018 were retrospectively analyzed, and they were classified into the S group (42 patients) and M group (42 patients). The patients in the S group received LNSS under SSRAC, while the patients in the M group received LNSS under MRAC. The duration of the operation, amount of intraoperative blood loss, intraoperative warm ischemia time, duration of postoperative hospital stay and positive rate of incisal edge; the serum creatinine and blood urea nitrogen values before and after the operation; and the occurrence rates of intraoperative and postoperative complications were compared.

**Results:**

All operations were completed smoothly. No patients had a positive incisal edge, and no patients were converted to MRAC during the operation. The duration of the operation and the amount of intraoperative blood loss increased in the S group compared with the M group. The differences were statistically significant (P <0.05). The differences in the intraoperative warm ischemia time, postoperative drainage and duration of postoperative hospital stay in both groups had no statistical significance (P >0.05). The differences in serum creatinine (SCr) and blood urea nitrogen (BUN) in both groups before the operation had no statistical significance (P >0.05). The SCr and BUN levels significantly increased 1 d and 1 m after the operation. The SCr and BUN levels 1 d and 1 m after the operation were significantly lower in the S group than in the M group, and the differences were statistically significant (P <0.05). The differences in the occurrence rates of intraoperative and postoperative complications in both groups had no statistical significance (P >0.05).

**Conclusion:**

SSRAC is a new renal artery clamping technology, and its curative effect on LNSS patients is significant. In addition, SSRAC has high safety and little influence on renal functions.

## INTRODUCTION

With the progression of laparoscopy technology, LNSS has been increasingly applied clinically. The tumor-specific survival rate and radical excision in LNSS patients have no obvious difference, and the long-term risk of death is obviously low. Thus,^1^,^2^ LNSS has become the first choice for the treatment of T1 localized renal tumors.^3^ After LNSS, renal functions are influenced by basic renal function, the number of nephrons retained and the warm ischemia time (WIT), and the WIT plays a key role.^4^ Renal artery clamping methods mainly include MRAC, segmental renal artery clamping, “zero” vessel clamping and selective renal artery clamping.^5^,^6^ Traditional nephron-sparing surgery mostly adopts MRAC for reducing the amount of intraoperative blood loss and improving the surgical field, which is beneficial for tumor excision and kidney recovery. However, this technology leads to warm ischemia of the kidney and adverse impacts on renal functions.^7^ In recent years, SSRAC has been increasingly applied in LNSS. SSRAC not only can achieve a tumor supply vessel clamping effect^8^ but also can effectively reduce patients’ renal WIT and reduce renal injury risk.^9^,^10^ In this study, LNSS under SSRAC was adopted to treat patients with T1 localized renal tumors and to evaluate its curative effect as well as renal function changes in the perioperative period.

Total 84 patients with T1 localized renal tumors who were admitted and treated from October 2017 to October 2018 were retrospectively analyzed, and they were classified into the S group (42 patients) and M group (42 patients). The patients in the S group received LNSS under SSRAC, while the patients in the M group received LNSS under MRAC. Inclusion criteria: (1) renal CT angiography (CTA) before the operation displayed grade II and grade III tumors with a blood supply from vessels of the kidney; (2) T1 phase, tumor diameter ≤4 cm (based on TNM staging of the American Joint Committee on Cancer); (3) R.E.N.A.L score 4~7; and (4) SCr was normal before the operation; after the operation, the patient was verified to have a renal tumor through pathology. Exclusion criteria: (1) solitary kidney; (2) severe heart, liver, lung and other organic lesions or other malignant tumors; (3) coagulation function and immune deficiencies; (4) hypertension and diabetes; and (5) combined regional lymphatic metastasis and distant metastasis. The differences in sex, age, BMI, tumor diameter, tumor phase, tumor position and R.E.N.A.L score in both groups had no statistical significance (P >0.05), as shown in Table-I.

**Table-I:** Clinical data of the patients (n/n) (X±S).

Group	No.	Sex (male/female)	Age (year)	BMI (kg/ m^-2^)	Maximum tumor diameter (cm)	Tumor phase (T1a/T1b)	Tumor position (left/right)	R.E.N.A.L (score)
S group M group	42	27/15	53.9±8.4	23.1±4.5	4.0±1.1	31/11	24/18	5.2±1.7
	42	30/12	54.3±8.5	23.7±5.2	4.0±1.2	29/13	28/14	5.1±1.5
t/ X^2^		0.491	0.217	0.401	0.296	0.233	0.808	1.391
P		>0.05	>0.05	>0.05	>0.05	>0.05	>0.05	>0.05

### Ethical Approval

The study was approved by the Institutional Ethics Committee (Date: 10 August 2019) of Jingzhou First People’s Hospital, and written informed consent was obtained from all participants

### Surgical Methods

The patients were placed in a lateral position. Four conventional holes were made at the waist, and the operation area of the posterior abdomen was established. The psoas major muscle was exposed; the adipose tissues outside the peritoneum and Gerota fascia were separated. The Gerota fascia was opened to separate the fat around the renal hilum, renal artery, front and back branches, and segmental renal artery. The presence of an ectopic artery at the position of the renal artery branch was judged based on the previous CTA examination. Then, the renal artery branch was dissected, and the renal tumor was separated. The scope of the tumor and the blood supply were confirmed, and the segmental renal artery was separated. A bulldog clamp was used to clamp the renal artery or the branch to completely block the blood supply to the artery of the tumor. Tracking the time was initiated during the clamping of the blood flow to the branch. The tumor was fully excised 0.5~1.0 cm from the edge of the tumor. Then, hemostasis and suturing of the wound surface were conducted. After the branch artery was opened, the clamping timing ended. The presence of active bleeding of the surface of the wound was observed. If active bleeding was present, suturing was initiated.

### M group

After the channel was established, the Gerota fascia was opened. The kidney was dissociated, and the renal artery was exposed. The renal artery was clamped to block the blood flow, and then the tracking of time was initiated. Next, the tumor was excised, and the surface of the wound was sutured. Next, a renal pedicle pincer was removed for the recovery of blood flow, and the renal artery clamping time ended. The tumor was removed, and the incision was closed.

### Monitoring indicators

(1) Indicators in the perioperative period: the duration of the operation, amount of intraoperative blood loss, warm ischemia time, postoperative drainage time, duration of postoperative hospital stay and positive rate of incisal edge; (2) SCr and BUN values were detected before the operation as well as 1 d and 1 m after the operation; and (3) occurrence rates of intraoperative and postoperative complications (hemorrhage, urine leakage, pulmonary infection, incisional wound infection and perirenal infection) were recorded.

### Statistical method

SPSS22.0 statistical software was used for data analysis. Enumeration data were expressed as the rate (%) and were tested with x^2^. Measurement data were expressed as (x±s). Independent-samples T test was used for the intergroup comparison, and intragroup comparison was conducted with paired *t* tests. P<0.05 indicated that the difference was statistically significant.

## RESULTS

No patients had a positive incisal edge, and no patients were converted to MRAC during the operation. The duration of the operation and the amount of intraoperative blood loss increased in the S group compared with the M group. The differences were statistically significant (P <0.05). The differences in the WIT, postoperative drainage and duration of postoperative hospital stay in both groups had no statistical significance (P >0.05), as shown in Table-II.

**Table-II:** Comparison of indicators in the perioperative period (X±S).

Group	No.	Duration of operation (h)	Intraoperative blood loss (ml)	WIT (min)	Postoperative drainage (ml)	Duration of hospital stay (d)
S group	42	102.3±26.2	103.9±12.5	25.1±6.6	189.4±19.5	6.6±1.5
M group	42	80.5±19.6	88.6±14.7	22.5±5.4	192.8±20.1	6.3±1.6
t		4.250	4.093	1.421	0.224	2.130
P		0.006	0.006	0.173	0.065	0.077

### Renal functions

The differences in serum creatinine (SCr) and blood urea nitrogen (BUN) before the operation in both groups had no statistical significance (P >0.05). SCr and BUN levels increased significantly 1 d and 1 m after the operation. The SCr and BUN levels 1 d and 1 m after the operation were significantly lower in the S group than in the M group, and the differences were statistically significant (P <0.05), as shown in Table-III.

**Table-III:** Comparison of renal function in the perioperative period (X±S).

Group	No.	SCr (µmol/L)			BUN (mmol/L)		
		Before operation	1 d after operation	1 m after operation	Before operation	1 d after operation	1 m after operation
S group	58	51.5±6.8	76.8±7.5*#	54.3±5.9*#	3.5±0.7	6.2±1.1*#	4.7±1.0*#
M group	42	54.5±7.2	97.2±8.9	79.6±9.2	3.6±0.8	4.7±1.0	3.8±1.0
t		1.531	3.821	4.750	0.558	5.943	5.371
P		0.156	0.010	<0.001	0.289	<0.001	<0.001

***Note:*** Intragroup comparison before the operation,

* P<0.05; comparison with M group, #<0.05.

### Complications

The differences in the occurrence rates of intraoperative and postoperative complications in both groups had no statistical significance (P >0.05), as shown in Table-IV.

**Table-IV:** Complication statistics (n/%).

Group	No.	Hemorrhage	Urine leakage	Perirenal hematoma	Incisional wound infection	Perirenal infection	Total
S group	42	0(0)	0(0)	1(2.38)	0(0)	0(0)	1(2.38)
M group	42	1(2.38)	1(2.38)	1(2.38)	1(2.38)	0(0)	4(9.52)
X^2^							1.914
P							>0.05

## DISCUSSION

Renal artery clamping is a conventional surgical method during LNSS for reducing renal bleeding, and this procedure inevitably causes warm ischemia injury to the kidney.^11^,^12^ Warm ischemia injury is a kind of ischemia reperfusion injury.^13^,^14^ The intraoperative renal WIT is an independent factor influencing renal function after nephron sparing surgery.^15^ Thus, how to shorten the WIT and reduce reperfusion injury as well as retain more functional nephrons under the precondition of ensuring a negative incisal edge have become key factors of LNSS.^16^ LNSS is only limited to a portion of the kidney and thus does not involve the whole kidney. This feature makes it possible to replace MRAC with SSRAC. In 2011, Gill et al.^17^ reported segmental renal artery partial nephrectomy related to renal tumor clamping for the first time and achieved a good effect. In the same year, Shao et al.^18^ reported that segmental renal artery clamping technology was used during nephron sparing surgery and proposed the concept of segmental renal artery clamping technology, which, to a certain degree, reduced the warm ischemia injury of the kidney during the operation. There are disputes about the application of SSRAC and MRAC during partial nephrectomy under a laparoscope. In this study, LNSS was conducted for 84 patients with T1 localized renal tumors under SSRAC and MRAC, and the clinical effects, advantages and disadvantages were compared.

It was found that the duration of the operation was obviously longer in the S group than in the M group, and the amount of intraoperative blood loss increased, which may be related to the fact that in the M group, the branched vessels supplying the tumor needed to be judged according to the preoperative CTA results. Renal artery clamping was carried out further, and the renal artery branch was clamped. Moreover, this study also found that the warm ischemia time, postoperative drainage time, duration of postoperative hospital stay, and intraoperative and postoperative complications did not increase in the S group compared with the M group. In addition, SSRAC had a small influence on the patients’ renal functions. This is because only the tumor-related renal artery branch was clamped in the S group, and there was still a blood supply for the other renal parenchyma, which could have reduced the warm ischemia injury to the normal renal tissues. In addition, the time limit of SSRAC was long so that the operator could excise the tumor cautiously and accurately. In addition, the positive rate of the incisal edge was reduced so that the surgical margin was thin. A large amount of normal nephrons could be retained, and the surface of the wound could be sutured. All these factors ensure the surgical effect to a certain degree and demonstrate a reduction in intraoperative and postoperative complications as well as the impact on renal function. Foreign reports have indicated that the glomerular filtration rate is obviously reduced after SSRAC compared with MRAC.^19^,^20^ This is similar to the result of this study.

## CONCLUSION

In conclusion, although SSRAC technology slightly increases the duration of operation and the amount of intraoperative blood loss, it successfully avoids complete renal ischemia and gains early renal function recovery compared with MRAC technology. Although SSRAC clamps the tumor-related renal artery branch and can lower the warm ischemia injury to the remaining renal tissues, SSRAC cannot completely achieve zero ischemia. Thus, improving the surgical skills of surgeons and shortening the duration of the operation are still required. This study still needs the support of data from a large sample size.

### Authors’ Contributions:

**YHL** **& JZ:** Designed this study and prepared this manuscript, are responsible for integrity of research.

**HTD** **&** **CML:** Collected and analyzed clinical data.

**ZYW:** Significantly revised this manuscript
